# The influence of experiential knowledge and societal perceptions on decision-making regarding non-invasive prenatal testing (NIPT)

**DOI:** 10.1186/s12884-020-03203-4

**Published:** 2020-10-19

**Authors:** Sophie Montgomery, Zaneta M. Thayer

**Affiliations:** Department of Anthropology, Dartmouth College, 6047 Silsby, Hanover, NH 03755 USA

**Keywords:** Non-invasive prenatal testing, Decision-making, Routinization, Experiential knowledge, Informed consent, Prenatal genetic testing

## Abstract

**Background:**

Non-invasive prenatal testing (NIPT) allows women to access genetic information about their fetuses without the physical risk inherent to prior testing methods. The advent of NIPT technology has led to concerns regarding the quality and process of informed consent, as a view of NIPT as “routine” could impair women’s considered approach when choosing to undergo testing. Prior studies evaluating NIPT decision-making have focused on the clinical encounter as the primary environment for acquisition of biomedical information and decision formation. While important, this conceptualization fails to consider how additional sources of knowledge, including embodied and empathetic experiential knowledge, shape perceptions of risk and the societal use of NIPT.

**Methods:**

In order to address this issue, qualitative, semi-structured interviews with 25 women who had been offered NIPT were performed. Participants came from a well-resourced, rural setting near a major academic medical center in the US. Women were categorized by NIPT use/non-use as well as whether their described decision-making process was perceived as making a significant decision requiring contemplation (“significant”) versus a rapid or immediate decision (“routinized”). A constructivist general inductive approach was used to explore themes in the data, develop a framework of NIPT decision-making, and compare the perceptions of women with differential decision-making processes and outcomes.

**Results:**

A framework for decision-making regarding NIPT was developed based on three emergent factors: perceptions of the societal use of NIPT, expected emotional impact of genetic information, and perceived utility of genetic information. Analysis revealed that perceptions of widespread use of NIPT, pervasive societal narratives of NIPT use as “forward-thinking,” and a perception of information as anxiety-relieving contributed to routinized uptake of NIPT. In contrast, women who displayed a lack of routinization expressed fewer stereotypes regarding the audience for NIPT and relied on communication with their social networks to consider how they might use the information provided by NIPT.

**Conclusions:**

The findings of this study reveal the societal narratives and perceptions that shape differential decision-making regarding NIPT in the U.S. context. Understanding and addressing these perceptions that influence NIPT decision-making, especially routinized uptake of NIPT, is important as the use and scope of this technology increases.

## Background

### Non-invasive prenatal testing technology

Non-invasive prenatal testing (NIPT) has significantly reshaped the options available to pregnant women to access genetic information about their fetuses since its initial introduction in 2011 [1]. NIPT technology analyzes cell-free fetal DNA (cffDNA) in maternal circulation to determine the risk of chromosomal abnormalities in the fetus [1]. NIPT is less invasive and can be performed earlier than amniocentesis and Chorionic Villi Sampling, as early as 9 to 10 weeks, and poses no physical risk beyond that of an already routine blood draw [1, 2].

NIPT is most often used for detection of aneuploidies in chromosomes 13, 18, and 21; however, an increasing amount of chromosomal abnormalities are being validated for testing [3]. Sensitivity rates of NIPT vary by aneuploidy, ranging from greater than 99% for trisomy 21, 97% for trisomy 18, and 91% for trisomy 13 [3, 4]. Although NIPT is a screening tool, rather than a diagnostic test, its relatively high sensitivity and specificity for trisomy 21 in particular “blurs the once-bright line between screening and diagnosis” [5], with evidence suggesting that women may misinterpret these screening results as diagnostic [6, 7]. NIPT is now commercially available in most of the world, although accessibility and cost vary [1, 8, 9]. Worldwide, NIPT is rarely covered as a “first-tier” test [9]. While some countries offer public funding for NIPT for women with high-risk pregnancies, in the United States, coverage for NIPT exists only through private insurance companies, which often cover the $600–800 cost for women with high-risk pregnancies [1]. In many low and middle income countries, NIPT is currently too expensive to allow for widespread access [1]. Women living in high-income countries are more likely to utilize NIPT due to higher income, greater genetic literacy, and a more robust infrastructure for implementation.

### Routinization and concerns about informed decision-making

The rapid uptake of NIPT has incited concern about its potential misuse. The implications of rapid NIPT uptake vary cross-culturally depending on factors such as the prevalence of sex-specific abortions and views on disability [1]. In the US and Western European context, where reproductive autonomy is highly valued, researchers have largely focused on the effect of NIPT on informed decision-making [1, 8, 10, 11].

The relative safety and non-invasiveness of NIPT may lead women to interpret NIPT usage as routine, expanding the threat of “routinization” [9, 10]. Routinization changes how women imagine and weigh their options, such that some women may view NIPT as “just another blood test” and uptake NIPT “without fully understanding its importance or implications” [9]. Thus, the technological ease of NIPT may obscure the significance of the decision to utilize it [12] and paradoxically undermine the ability for women to make autonomous, informed reproductive choices [9]. Studies have corroborated this concern, finding that both women and practitioners view the consent process for NIPT differently than invasive prenatal testing, and that women are more likely to make uninformed choices regarding NIPT [7, 11]. Addressing concerns about routinization is increasingly important as the scope of NIPT sequencing expands [13] and the degradation of informed decision-making leaves women emotionally unprepared for the results they may receive [5, 14].

### Influence of experiential knowledge and societal perceptions on decision-making

Prior studies investigating NIPT-related decision-making have primarily focused on the clinical interaction as the environment for acquisition and application of biomedical knowledge to make informed decisions [15–17]; for exceptions see [8, 18, 19]. However, analysis of decision-making must consider “discourses beyond the biomedical domain” [18] since a significant portion of the decision-making process occurs outside of the clinical encounter [20] and may rely upon perceived societal values and experiential knowledge regarding NIPT.

In terms of the potential influence of perceived societal values on NIPT decision-making, prior investigations have noted how technological advancements in prenatal technology are accompanied by a societal expectation to uptake such technologies in order to avoid what are increasingly seen as avoidable consequences [21–23]. Maternal responsibility thus shifts from caretaker to “quality control” [23, 24]. Such societal perceptions regarding the use and implications of NIPT may shape how women approach the decision of whether or not to use it, and are therefore important to consider when evaluating NIPT decision-making.

In addition, NIPT decision-making may be influenced by experiential knowledge. Experiential knowledge takes two forms, embodied and empathetic. Embodied knowledge is defined as knowledge obtained through personal, bodily experience (e.g. prior pregnancy), whereas empathetic knowledge is gained through exposure to the experiences of family and friends [25]. Etchegary et al. [25] and Browner and Press [26] have explored how experiential knowledge influences prenatal decision-making; however, limited work has applied this theoretical lens among women considering NIPT (for exceptions see [19, 22, 27]).

Personal characteristics, such as age, health history, and health status contribute to embodied knowledge [25, 28]. Positive health status has been identified by women “as a factor that would decrease risk”, whereas a personal history of miscarriage or infertility leads women to lose “a sense of control over their bodies” and perceive greater risk [28]. Thus, embodied forms of knowledge inform subjective risk perception, which is not necessarily associated with actual risk [29], but may significantly shape NIPT decision-making. In addition to personal characteristics and experiences, vicarious knowledge about the experiences of friends, colleagues, and family contributes to empathetic knowledge that shapes perceptions of NIPT and may inform subsequent decision-making [25].

While theoretical foundations regarding experiential knowledge and perceived societal values have highlighted important influences on prenatal testing decisions [5, 25, 26], many of these theoretical foundations have not been directly explored among women offered NIPT. Additionally, prior studies investigating perceptions of, and knowledge about, NIPT among women considering its use have not evaluated differences in the frameworks used by women who do or do not uptake NIPT or who display routinized as opposed to significant decision-making [19]. Understanding decision-making frameworks among women from these different categories will help inform strategies for avoiding routinized decision-making regarding NIPT.

### Aims

This study sought to (1) elucidate whether there are decision-making frameworks that characterize maternal decision-making regarding the uptake of NIPT; and (2) to explore how such frameworks may be shaped by background knowledge about NIPT, perceived societal values, and experiential knowledge, both embodied and empathetic. This study also aimed to compare decision-making between women with different decisional processes (routinized vs. significant consideration) and outcomes (use vs. non-use of NIPT).

## Methods

In order to assess the decision-making processes regarding NIPT, semi-structured, qualitative interviews were conducted with women who had been offered NIPT. This study was performed from a constructivist point of view, based on the assumption that meanings and knowledge underlying NIPT decision-making are socially constructed phenomena. Researcher SM, a female undergraduate anthropology major with experience conducting semi-structured qualitative interviews, recruited and interviewed the participants along with guidance and input by ZM, PhD in anthropology and Assistant Professor of Anthropology.

This study was conducted in a well-resourced, rural setting near a major academic medical center in the Northeastern United States, providing a robust infrastructure for access to NIPT. Potential participants were recruited with flyers and Facebook postings in community groups. Facebook posts and flyers communicated that the interviews would be conducted by a female undergraduate student exploring how women make decisions about non-invasive prenatal testing. No prior relationship was established between the researchers and potential study participants.

Women interested in the study emailed or called SM to be screened for eligibility and set up an interview time and location. Eligibility was determined by residency in the rural area surrounding a major academic research hospital and by having a current or recent pregnancy (with a child 2 years or younger). This cut point in child age was used to ensure that mothers involved in the study were pregnant recently enough to have been offered NIPT and to recall their decision-making process.

Participants first met the researcher (SM) face-to-face at the interview location. The interview was conducted at whatever location was preferred by the study participants that offered privacy. Most interviews were conducted in local coffee shops, participant’s homes, or local libraries. Participants were invited to bring children if necessary. Some participants had young children with them during the study interview. Interviews lasted 20 mins to 1 h and an interview guide [See Additional file 1] was used to guide conversations, with questions asking about all phases of decision-making including prior knowledge, interaction with a provider, sources of knowledge regarding NIPT, and the ultimate decision. These questions served as a starting point and general outline for the discussion.

Interviews were conducted between September 2018 to February 2019. Twenty-five interviews were conducted, and recruitment was terminated when saturation was reached (no new themes were identified). None of the participants refused to participate or dropped out of the study. Interviews were recorded with participant knowledge and consent for subsequent transcription by the study author SM. Brief field notes with observations and reflections were written by the interviewer after each interview. Interview transcripts were uploaded to NVivo 12 software for management.

A general inductive approach was used to evaluate the decision-making process regarding NIPT. The general inductive approach is meant to assist in the development of models about “the underlying structures of experiences” and allow for summarizing of these experiences in a brief, summary format [30]. This approach is often used in health and social sciences [30].

Interview transcripts were read thoroughly and initial in vivo coding was applied using NVivo. Codes represented distinct factors that informed decision-making and relevant perceptions. After initial open coding was applied to explore factors, these codes were subsequently grouped into categories. The transcripts were re-read multiple times and codes were reorganized, rewritten (focused coding) and grouped into categories to explore factors affecting decision-making and differential perceptions. Comprehensive lists of quotes grouped under categories were compiled.

In order to evaluate how routinization affects decision-making among women in this sample, women’s descriptions of their decision-making processes were evaluated based on descriptions of routinization in the literature as shaping perceptions of NIPT as “just another blood test” and promoting uptake with minimal or no consideration [9]. The quality of decision-making (routinized vs. significant decision-making) was constructed based on women’s expressed description of their perception of the significance of the decision-making process. Women who expressed that they did not consider the uptake of NIPT to be a significant decision and either decided immediately or entered the appointment already knowing their intentions were categorized as having “routinized” uptake or “automatic dismissal”, respectively, depending on whether or not they used NIPT. Women who perceived the NIPT decision as a significant decision and described contemplation of NIPT uptake or non-uptake before making their decision were categorized as having “significant decision-making.”

Additionally, in order to evaluate how the coded factors shape differential decision-making, women were grouped by the quality (routinized vs. significant) and outcome of decision-making (NIPT use or non-use). Interview transcripts were re-read and coded factors were compared across the different groups of women with differential outcomes and decision-making processes. Coding was restructured and each category was continuously revised for inconsistencies. The study authors collaborated to discuss emerging frameworks for decision-making and come to a consensus regarding categorizations and patterns. A framework was constructed based on the patterns found across groups among coded themes, and key quotes were identified as representing core themes.

## Results

### Participants characteristics and uptake of non-invasive prenatal testing

This study consisted of 25 women who were between the ages of 27 and 39 during their current or most recent pregnancy. For five participants, the reported pregnancy was their first pregnancy; nine participants had one pregnancy prior to the discussed pregnancy; 11 women had two or more previous pregnancies. Most of the women in this sample (*n* = 18) used NIPT technology during their current or most recent pregnancy. Approximately half of the women in this sample (*n* = 13), and most of the women who used NIPT (13 of the 18 women who used NIPT) displayed routinized uptake.

Fewer than one third (*n* = 8) of women were 35 or older and therefore clinically defined as advanced maternal age (AMA). The majority of participants were married (*n* = 23), and most had earned a bachelor’s degree or higher (*n* = 21). The women in this sample were predominantly white (*n* = 24) and of high socioeconomic status (21 participants with household income above $90,000). Additionally, three participants self-reported family history of genetic disorders (specific disorders were not described). Ten women reported a history of miscarriage, and one participant reported being previously told she was infertile.

### Background knowledge about NIPT

Almost all of the women described a vague understanding of prenatal genetic testing before they encountered the decision of whether to use NIPT. Due to the novelty of NIPT technology, most of the prior knowledge women had acquired about prenatal genetic testing through their social networks was about amniocentesis:

“That’s the only thing I’d ever heard was like a needle will go through your belly. That’s pretty much all that I ever knew about genetic testing prior to being pregnant.” (Participant 17; non-AMA, personal history of miscarriage; family history of genetic disorders).

Thus, stories from mothers, aunts, and friends informed empathetic experiential knowledge regarding amniocentesis, and these perceptions of amniocentesis were largely negative and often accompanied by a vivid image of a “big needle through the uterus”. Based on this background, NIPT was evaluated in comparison to amniocentesis, rather than exclusively on its own terms:

“I think when I thought genetic testing, that was kind of the thing that I represented in my mind. And I guess I was happy to know that there were noninvasive methods to use.” (Participant 12; non-AMA; used NIPT)

This basis for contemplation of NIPT emphasizes its non-invasive nature compared to amniocentesis and thus frames the technology as especially appealing as a high benefit procedure without the risk of miscarriage present in earlier genetic testing technologies.

### Frameworks for NIPT decision-making

Thematic analysis of interview transcripts was conducted to identify a framework by which women approach decision-making regarding the uptake of NIPT. Women’s evaluations and perceptions of three factors were identified as instrumental in decision-making:

1. Perceived societal use of non-invasive prenatal testing
  Women’s perceptions of how often NIPT is used, who uses it and why, as well as how they categorize themselves in relation to these classifications informs decision-making.
2. Expected emotional impact of genetic information
  Women’s forecasting of how the information provided by NIPT will affect them emotionally also factors into decision-making. Women may perceive information as emotionally neutral, anxiety-inducing, or anxiety-relieving.
3. Perceived utility of genetic information
  Women’s evaluation of how the information provided by NIPT may be of use to them shapes decision-making. Information may be seen as useful due to its ability to allow parents to prepare for having a child with chromosomal abnormalities. Conversely, information may be seen as useful in terms of how it may inform termination decisions.

Women were categorized based on whether they used NIPT as well as whether they considered the decision-making process to be significant (routinized vs. significant). Characteristics of women in each of these categories were considered as well as how women in each of these categories differ along the three factors outlined above. Women who displayed non-routinized decision-making, regardless of NIPT use, were analyzed together (Group 3) and compared to women displaying routinized use (Group 1) and routinized non-use (Group 2) of NIPT. Results of this analysis are presented below, with a summary presented in Table 1.

**Table 1:** Frameworks for Differential Decision-Making. The table below summarizes the perceptions of women in each category of decision-making of the three factors in the framework outlined above

	Routinized	Non-Routinized
**Uptake**	**Group 1: Routinized Uptake of NIPT** (*n* = 13) 1. Common usage for science-friendly people 2. Information as anxiety-relieving or emotionally neutral 3. Utility is preparing for a child with a disability	**Group 3: Significant Consideration of NIPT Resulting in Uptake or Non-Use** (*n* = 8) 1. Mixed usage; individual choice based on circumstances 2. Information as anxiety-relieving or emotionally neutral 3. Utility is informing termination decisions
**Non-use**	**Group 2: Automatic Dismissal of NIPT** (*n* = 4) 1. Only use if specific risk 2. Information as anxiety-inducing 3. Utility is informing termination decisions (not willing to terminate)	

### Group 1: routinized uptake of NIPT

Women with routinized uptake did not perceive the decision of whether to undergo prenatal genetic testing as significant, and many did not consider it to be a decision at all. As two participants stated:

“I think I just assumed we would [do the test] because it’s not like super invasive.” (Participant 11; non-AMA).

“I felt like yeah, since it seems like it was not invasive, you know, why not get this information” (Participant 23; non-AMA).

Approximately half of the women in the study sample demonstrated routinized uptake of NIPT (*n* = 13). Most of the women in the routinized uptake category were below age 35 (*n* = 9) and did not self-report a family history of genetic disorders. Women in this group characterized the factors in the aforementioned decision-making framework in the following ways:

1. Perceived societal use of non-invasive prenatal testing
  Women with routinized uptake of NIPT perceived common usage of NIPT technology. This perception was bolstered by stories of friends and family who used the test. Women with pregnancies in this category did not discuss NIPT much with their network as they were considering whether or not to use the test, with many only speaking to their partner and provider about the decision. Their perceptions of NIPT were therefore primarily shaped by discussions about prenatal genetic testing that took place before their pregnancy. For example, women who perceived that others in their social network were using the test often expressed that this increased their comfort with NIPT:
  “Well, my sister had done the testing, the blood test… I felt okay to do the blood tests, that type of testing because she did it, so I assumed it was safe.” (Participant 10; non-AMA; family history of genetic disorders).
  “I think given the societal comfort with that kind of screening, it probably made me feel more comfortable even on an unconscious level about having the testing done.” (Participant 2; non-AMA).

This perception of common usage normalized uptake of NIPT and increased comfort with the technology. Thus, conversations with other women undergoing testing served as a form of empathetic experiential knowledge informing women’s perceptions of the test and its societal use.

In addition to ideas about how often NIPT is used, women displaying routinized uptake also expressed common perceptions regarding who uses NIPT technology. In describing their own usage of the technology, women in this routinized category characterized themselves as science-friendly and “pro-information”. They emphasized this personal orientation as a reason for their uptake of the test and implied that denying use of NIPT would be motivated by an anti-science or religiously-oriented approach:

“I try to surround myself with science people and with information about new breakthroughs in science … and so, like if we’re counting all of those groups, kind of society at large for me, um, I think that would all be very pro information and like have as much information as we can.” (Participant 7; non-AMA; personal history of miscarriage).

“I work in a culture that is science-friendly. And so tends to be, you know, it’s data-driven and I know the differences between screening and a definitive test, you know. And so I think in my most immediate cultural circles I felt encouraged.” (Participant 6; AMA).

One participant, while not part of the routinized uptake group, articulated this association of NIPT use with pro-science tendencies as follows:

“I felt like in society the general measure of things was kind of, it was treated as like, if you were scientific and forward-thinking you were in support of these things and if you are maybe from a religious community or didn’t understand science, then you were against them and there was a sort of polarization that I felt like happened along those lines.” (Participant 3; AMA)

2. Expected emotional impact of genetic information
  Women in the routinized use category tended to anticipate that the information provided by genetic testing would be anxiety-relieving or emotionally neutral. Anxiety relief was cited as a major reason for pursuing testing by many:

“The physician, so in that one appointment she brought it up and we already knew that we wanted to do it. Not that we have any risks, you know, I mean but just for peace of mind.” (Participant 19; non-AMA).

Some of the women who had experienced miscarriages prior to their current pregnancy expressed a greater desire for reassurance due to that experience:

“It was fairly recently after my miscarriage when I had the second pregnancy. So I just wanted that extra assurance.” (Participant 25; non-AMA; personal history of miscarriage).

Thus, prior experience of miscarriage served as a form of embodied experiential knowledge that affected the desire for reassurance.

For those who saw genetic information as emotionally neutral, the decision to obtain information was seen as a precursor to the actual decision of what to do with it. These women cited an emphasis on informed decision-making and a desire to always have more information rather than less:

“I just was like, of course we’re going to do it because, because um, when we have information, we have data, so we pretty much decided and just went with it” (Participant 21; non-AMA; personal history of miscarriage).

“I like being informed. I like making informed choices. I didn’t know exactly what we would necessarily do with the information, but I knew that we wanted it” (Participant 6; AMA).

This emphasis on having as much information as possible aligned with the science-friendly, forward-thinking self-perception of women displaying routinized uptake.

3. Perceived utility of genetic information
  Women in this category predominantly characterized the perceived utility of genetic information as its ability to allow parents to prepare for having a child with a chromosomal abnormality. Many women expressed positive views of disability and an intention to continue their pregnancy regardless of NIPT results. Given this intention, NIPT offered a way to be prepared:
  “Like if it’s trisomy whatever and baby’s not going to survive past the first week, then you know, just mentally preparing for that. And then of course for Downs. We would just again prepare for that. So at the time of the birth you can really celebrate, even though it’s going to be difficult, you can celebrate the birth and not be focused on like this new, very devastating diagnosis. You can kind of deal with it in advance, prepare for it mentally. So when the baby comes you can really just still celebrate. Yeah, so I think it was just preparing.” (Participant 16; AMA).

Overall, the decision-making process regarding NIPT use for nearly half of the women in this study sample was routinized. For these women, the decision of whether to undergo NIPT was seen as, to quote one participant, a “no-brainer” due to perceived common usage of NIPT among science-friendly people, an expectation of receiving information that is anxiety-relieving or emotionally neutral, and the ability of this information to help families prepare for a child with chromosomal abnormalities.

### Group 2: automatic dismissal of NIPT

Women who did not use NIPT technology and who did not significantly consider its use were classified as part of Group 2. The decision-making process of these women was therefore considered routinized in a different sense. Similar to women who displayed routinized uptake of NIPT, the women in Group 2 did not perceive the decision of whether to undergo prenatal genetic testing as significant. Additionally, similar to the women displaying routinized uptake, women who dismissed the use of NIPT during their pregnancy made the decision quickly and usually discussed it only briefly with their partner and provider.

There were four women in the sample that could be categorized as part of this group. These women were all below 35 and therefore not considered AMA. Only one woman in this group had a history of miscarriage, and none of the women in this group had a self-reported family history of genetic disorders. Women in this group characterized the factors in the NIPT decision-making framework in the following ways:

1. Perceived societal use of non-invasive prenatal testing
  Similar to the women displaying routinized uptake, women who immediately dismissed the use of NIPT during their pregnancy perceived widespread use of NIPT. However, unlike women with routinized uptake, the women in Group 2 thought that the use of NIPT was specifically meant for women considered “high risk” due to factors like advanced maternal age or family history of genetic disorders. Therefore, women who automatically dismissed NIPT did not consider NIPT use largely because they perceived that the test was meant only for a “high risk” category which they did not see themselves as part of:
  “I always viewed it as like genetic testing is something that you get if you know it runs in the family… or if you are older than 35 or in your forties and you’re pregnant, the doctor is more likely to be concerned.” (Participant 1; non-AMA).
  “I felt, having done it before, having had a healthy child before, I didn’t need that degree of reassurance maybe the second time… I seem to be able to carry them to full term no problem.” (Participant 23; non-AMA).

In sum, women’s perceptions of their individual risk, which is likely shaped by experiential knowledge as well as the clinical encounter, led them to perceive their pregnancies as “low risk” and therefore to believe that they were not part of the target NIPT audience.

2. Expected emotional impact of genetic information
  The genetic information made available via NIPT was characterized by women in Group 2 as anxiety-inducing. The potential anxiety of receiving the information was cited as a deterrent to pursuing testing:
  “But it’s scary because if you know that there’s nothing you can do, at least with my personality, I’m just more likely to just worry and stew be like, oh, was there something that I could do? Um, whereas if I didn’t know, like I would just deliver the baby and yes, I would get the news that the baby had, you know, x or y condition. Um, and then I would start dealing with it right then and there instead of having the worry of baby can be healthy, the baby might not be healthy and so it’s just kind of the worry” (Participant 1; non-AMA).

This characterization of biomedical information as anxiety-inducing contrasts with women in Group 1, who primarily viewed this information as anxiety-relieving or neutral. This is particularly interesting given women in Group 2’s self-perception as “low risk,” and indicates that the characterization of biomedical information as anxiety-inducing is not due to any heightened personal risk but rather a general response to biomedical testing that these women perceive as personally unnecessary.

3. Perceived utility of genetic information
  Women in Group 2 saw the utility of genetic information in its ability to inform termination decisions. Therefore, according to this view, testing is done by parents interested in pursuing termination in response to test results indicating chromosomal abnormalities. Based on this perception, their own intention to carry the pregnancy to term regardless of NIPT results contributed to their lack of interest in NIPT:
  “We felt that no matter what we were going to carry the pregnancy full term. So it sort of didn’t matter if you will.” (Participant 14; non-AMA).
  “We just decided that even if we found out anything we probably wouldn’t have done, like we wouldn’t have aborted or anything like that if there was a problem.” (Participant 8; non-AMA; personal history of miscarriage).

This view directly contrasts with that of women displaying routinized uptake of NIPT (Group 1), for whom genetic information is not for informing termination decisions, but rather has utility in preparing parents for having a child with special needs.

Overall, the women who displayed automatic dismissal of NIPT characterized the information provided by NIPT as anxiety-inducing and understood its utility to be for those with specific risk who would potentially terminate a pregnancy. Based on these perceptions, the women in this category did not consider using NIPT and little consideration was necessary to decide to decline testing.

### Group 3: significant consideration of NIPT resulting in uptake or non-use

About a third of women (*n* = 8) in the study significantly considered the uptake of NIPT. In five instances, the women ultimately decided to use NIPT, while in three they did not. The women in these two categories are considered together in this analysis due to similar characteristics of their decision-making processes and perceptions. Almost all of these women (*n* = 7) were 35 or older, had a prior screening result indicating possible fetal abnormalities, or had a self-reported family history of genetic disorders. Women in this category approached the decision-making process using the framework described above with distinct perceptions of each of the three factors:

1. Perceived societal use of non-invasive prenatal testing
  Women who significantly considered the use of NIPT acknowledged widespread societal use of the test. However, unlike women in Group 1, they did not associate NIPT use with a forward-thinking mindset or assume that most women use testing. Additionally, unlike women who dismissed NIPT, women in this group did not assume that NIPT is only for use by women with specific high risk. Instead, these women described mixed use of NIPT societally and pointed towards the potential positives of testing for some women, while also noting the importance of individual choice based on personal preferences:
  “So I think it certainly is something that I think some certain people really go to and others shy away from it. I think we fell right in that middle ground seeing where it’s useful, but also not doing it just because. So I’m certainly glad it exists even though we didn’t partake in it.” (Participant 17; non-AMA; personal history of miscarriage; family history of genetic disorders; did not use NIPT).

When asked to consider how their peer networks or broader societal influences may have shaped their perceptions of NIPT and decision-making, many of the women in this group asserted that they did not feel pressured to decide one way or another and that the decision was ultimately a personal one:

“But personally, I felt really secure with my choice and with friends and other people.. . I think they were really all very like, yeah, whatever you feel more comfortable with is fine.. .I feel like people were like, oh, what are you going to do? And that was it. So I never really had any feel about what other people’s perceptions of the tests or if we should or shouldn’t do them.” (Participant 17; non-AMA; personal history of miscarriage; family history of genetic disorders; did not use NIPT).

“I must not really care that much about what society thinks. Maybe it’s just more, okay, well how does this affect me?” (Participant 24; AMA; used NIPT).

Women in this group thus conceptualized NIPT as a powerful tool which may be useful in some situations, but which each family must consider within the context of a pregnancy to determine if it is right for them. This perception of NIPT use formed the foundation for a significant decision-making process as women considered their own use.

In terms of how women developed perceptions of societal usage, women in Group 3 relied to a greater extent on vicarious experiences of others in their network as a form of knowledge to inform decision-making. In talking to friends, family, and colleagues, or reading blogs online, women collected stories of others’ experiences as scenarios to think through:

“I also read like chats on forums. So there are a lot of conversations out there where moms would try and make this decision. Um, and I read those conversations to get a better feel for this, like the cascade of events that happens after, because that’s the main question that I had.. .. I just like read people’s sort of thought processes and um, read about people’s experiences either getting a clean test or getting a test results back that indicated there was a problem.” (Participant 3; AMA; did not use NIPT).

2. Expected emotional impact of genetic information
  Among women who significantly considered the use of NIPT, women who ultimately decided to use NIPT differed from women who did not in terms of their characterizations of information as anxiety-inducing or anxiety-relieving. As one woman who decided to use NIPT after significant consideration stated:
  “It was reassurance, just knowing that, okay, he’s got less than one in 10,000 chance of being Down syndrome. So, um, it’s still because it’s, there’s still a chance, there’s still a little bit of a nagging thing in the back of your head, but it’s a little tiny chance. So you can kind of feel better about it. You’re not going blind.” (Participant 22; previously told infertile; prior screening result indicating possible fetal abnormality; used NIPT).

In contrast, a participant who decided to not use NIPT after significant consideration explained the potential stress of receiving genetic information indicating an abnormality:

“If my baby’s born with this, do I really want to panic my whole pregnancy and not enjoy it knowing that my baby is going to come out, you know, needing extra help or do I just grin and bear it and when he’s born, if he needs that help then we’ll get into that help? Like that’s where I was like really stuck out.” (Participant 4; non-AMA; personal history of miscarriage; family history of genetic disorders; did not use NIPT).

3. Perceived utility of genetic information
  Similar to women in Group 2, women in Group 3 perceived the utility of NIPT technology as its ability to inform termination decisions. The decision about whether to use the test therefore became a decision about whether one would be willing to have a child with a chromosomal abnormality. Based on this understanding of the utility of NIPT, women in this group who ultimately decided to uptake NIPT did so citing a willingness to potentially terminate a fetus, while women who did not use NIPT did so due to their stated decision to carry to term regardless of NIPT results:
  “You know, for me I felt like, I guess I wasn’t sure if I wanted to have a child with a disability and if there was an option to test for that and to terminate the pregnancy that, that would be my preference.” (Participant 13; AMA; used NIPT).
  “Then my doctor told me it was past the date for an abortion. So if you did have the baby and it did have a genetic disability, I’m already past the date, so that’s when I was like, I’m not going to do it because I’m not going to worry.” (Participant 4; non-AMA; personal history of miscarriage; family history of genetic disorders; did not use NIPT).

One of the participants in the study described how her use of NIPT changed across two pregnancies. She significantly considered NIPT for both pregnancies, but came to different decisions for each, declining use of NIPT for her first pregnancy but deciding to use it for her second. When describing her change in decision, she explained that her willingness to terminate was altered due to life circumstances, and this ultimately led her to use NIPT for the second pregnancy:

“My first pregnancy, we declined most testing with that pregnancy. I think our thoughts were for all we know this might be our only opportunity to have a child and there are certain disabilities that we’re okay with being parents to this child, like we’re up for that challenge.. . If we were to have a child with disabilities now, we’re not sure that we would be able to cope with that. Like it would really be a huge transformation of our lives and in a way it would be taking away a lot of time and resources to our older daughter. Um, so that was why we were sure that we wanted to do testing this time around” (Participant 13; AMA; used NIPT).

Overall, women in this group who did use testing varied in terms of what conditions they deemed significant enough to warrant termination; however, the underlying assessment of NIPT as a means of informing termination decisions remained consistent, and women may make different decisions across their reproductive careers due to altering circumstances which affect their willingness to terminate a pregnancy.

## Discussion

In exploring NIPT decision-making, we found similarities among women exhibiting non-routinization of NIPT, regardless of NIPT use or non-use decision, and differences between women exhibiting routinization depending on use or non-use.

### Perceptions of societal use and values

Our findings build upon prior studies that have investigated patient attitudes and knowledge about NIPT’s safety and accuracy [31] to highlight how perceptions of societal use of NIPT influence decision-making. Women’s perceptions of the frequency of NIPT use, as well as who uses this technology, shaped whether they viewed themselves as part of the intended audience for NIPT. For example, women who displayed automatic dismissal of NIPT did so due to their perception that NIPT is exclusively for those who have specific risk. Their own self-perception as low-risk and therefore not part of what they saw as the intended audience of NIPT led to an automatic dismissal of its use. In contrast, women who displayed routinized uptake of NIPT perceived its usage to be common among all women and thus their use of NIPT required minimal consideration. Women for whom the decision of whether to use NIPT was significant (non-routinized) did not describe a specifically prescribed audience for the test. Instead, they emphasized the personal nature of the decision based on individual preferences.

In terms of the impact of perceived societal values on decision-making, women who displayed routinized uptake of NIPT expressed the perception that use of the technology is associated with being science-friendly and forward-thinking. This perceived expectation of NIPT use by those who are “science-friendly” aligns with analyses by Rapp and McCoyd about the increasing societal expectation to use NIPT [21, 23].

These findings of the perceptions that shape routinized decision-making suggest that societal discussion and clinician framing of NIPT should aim to emphasize that although it is a powerful technology, NIPT uptake requires individual consideration, as women who perceived the decision as significant described. Lessening routinized uptake also requires countering societal narratives of technology as always progressive and the non-use of NIPT technology as regressive.

### Expected emotional impact and perceived utility of genetic information

Women who did not use NIPT, whether via automatic dismissal or after significant consideration, conceptualized the genetic information as anxiety-inducing. In contrast, women who used NIPT, whether via routinized uptake or after significant consideration, described the expected emotional impact of information as reassuring. Lewis et al. similarly described how women deciding to use NIPT did so seeking “peace of mind” and “control” [31]. Even if the results were undesirable, women in Lewis’ study described that the “increased sense of control outweighed any anxiety the information generated” [31]. Thus, our findings corroborated Lewis et al.’s, revealing that the conceptualization of genetic information as anxiety-relieving is a central component of the decision to use NIPT.

Women displaying routinized uptake were also likely to describe the information as emotionally neutral and emphasized their desire to make “informed decisions”, regardless of whether they have an idea of how they may use the information. These same women displaying routinized uptake emphasized the utility of genetic information as allowing them to prepare for having a child with a disability. Thus, their use of NIPT centered around gaining knowledge. This perception stood in contrast to that of women displaying automatic dismissal or significant decision-making, who saw the utility of the information as informing termination decisions. In this case, the decision of whether to uptake NIPT becomes entangled with the decision of what to do with the information and whether one is willing to terminate.

Since women displaying routinized uptake did not consider this test within the context of termination, it could result in women being unprepared to receive results indicating a chromosomal abnormality. Mozersky describes this detachment of the decision to use NIPT and the decision of how to use that information as deferred ethical thinking and argues that women’s desire for reassurance may come at the cost of women being unprepared for the results they receive [14]. Our results suggest that the women who saw themselves as the most forward-thinking actually displayed the most routinized uptake, aligning with concerns expressed by the medical community and rendering them vulnerable to the shock and distress that Mozersky describes.

### Embodied and empathetic forms of knowledge

Etchegary et al. described how experiential knowledge, both embodied and empathetic, “plays a pivotal role in [women’s] thinking about the value of prenatal tests” [25]. NIPT was introduced to the market after Etchegary et al’s manuscript, and much of the literature surrounding NIPT use has focused on women’s understanding and perceptions of the test itself, including its safety and accuracy [31]. However, technical knowledge of NIPT cannot explain decision-making on its own, since women give “their own meaning to these aspects in accordance with their lives” [25]. Our findings build on prior research regarding NIPT decision-making by applying the lens of experiential knowledge to this new technology.

Embodied experiential knowledge shapes decision-making by influencing the level of desire women express for reassurance. One of the most relevant bodily experiences informing prenatal testing decisions is prior pregnancy. Women with a personal history of miscarriage often cited this experience as a reason for pursuing testing due to an increased desire for reassurance. In contrast, some women mentioned that prior healthy pregnancies increased their trust in their bodies, leading them to feel a decreased desire for reassurance, and therefore they did not pursue testing.

In terms of empathetic experiential knowledge and its impact on decision-making, many women reported hearing stories of their mothers and aunts using amniocentesis, and these stories informed experiential knowledge about this method as invasive and unpleasant. This background knowledge shaped views of NIPT as relatively non-invasive. Additionally, although many women displaying routinized uptake or automatic dismissal of NIPT did not perceive the decision to be significant and therefore did not consult their social networks beyond their partner and provider while deciding, conversations and stories prior to pregnancy contributed to their perceptions of NIPT. For example, women displaying routinized uptake of NIPT perceived common usage of NIPT due to their prior conversations with people in their social networks. In contrast, women who saw the decision of whether to uptake NIPT as significant consulted their social networks and searched online to a greater degree while making their decision and used the scenarios they learned about to think through how they may use the information provided by NIPT.

An emphasis on the impact of embodied and empathetic experiential knowledge reveals how decision-making frameworks change as these forms of knowledge change. Many women expressed that the embodied knowledge gained from prior pregnancy experiences (whether ending in miscarriage or a healthy child) affected decision-making in subsequent pregnancies. Thus, women do not approach decisions with a fixed set of “values” and “beliefs” as prior studies posit [15, 32]; instead, decision-making is a dynamic process that may change between pregnancies based on changing experiential knowledge and circumstances.

### Influence of providers on decision-making

Concerns have arisen regarding the potential influence of providers in encouraging routinization due to the patient-provider power imbalance and the possibility of women feeling pressured to uptake NIPT [18, 21, 28]. When asked specifically about provider influence, women across all groups in this study emphasized the neutrality of their providers and felt that they would be supported in whatever decision they made. Hence, provider influence was not included in the framework described above since this was not a significant factor for women in this sample. While some clinical environments may result in women feeling pressured to uptake NIPT, the lack of clinician pressure experienced by women in this study along with their routinized uptake of NIPT suggests that routinization can be shaped by forces outside of the clinical encounter.

When asked about clinician influence, many of the women in this study attributed their clinician’s neutrality to their use of a midwife for care during pregnancy. The majority of the women in this sample (*n* = 18) used midwives, and it is possible that the perceived provider neutrality is related to the midwifery approach. When describing why they chose to use a midwife, many women described themselves as desiring a more “holistic” approach to pregnancy [33] and were largely critical of what has been referred to as the biomedicalization of pregnancy and childbirth [34]. Women cited their desire for a more “natural” and less “invasive” pregnancy experience as the motivation for seeking midwifery care. At the same time, the majority of the women in this study used NIPT, with half fitting into the routinized uptake category. While prior studies have expressed concern for clinicians pressuring women to use NIPT in highly medicalized environments [20, 27], the findings of this study suggest that routinization also occurs among women who identify as adopting a more “holistic” mindset and who use midwife providers. The marketing of NIPT as a “non-invasive” test, especially in contrast to amniocentesis, may make it more palatable to these women. Thus, while many of these women adopt aspects of a holistic paradigm [32], routinized uptake of NIPT is still pervasive, perhaps reflecting the societal privileging of data and information.

This seemingly contradictory uptake of NIPT among women who identify as adopting a more “holistic” mindset may also reflect the specific demographics of the women in this sample. As a highly educated sample, these women may be more likely to feel the need to align themselves with a “pro-science” mindset. Thus, aspects of identity may shape the extent to which women feel that they should use NIPT, and further research should aim to explore this framework among demographically diverse groups to explore how these patterns may differ.

### Limitations

There are several limitations of this study that should be addressed in future work. Women described their decision-making experiences within the last two to 3 years, and these retrospective descriptions may be biased by the outcome of the pregnancy.

This study did not inquire about insurance coverage specifically; evaluation of insurance-coverage and its influence on decision-making should be evaluated in more detail in future studies. Further, we did not systematically collect information on family history of genetic disorders or presence of conditions detected by NIPT in prior pregnancies; instead, this topic came up as spontaneously during several interviews. Further exploration and systematic evaluation of these factors in future work will allow for better assessment of how they shape the embodied experience that informs decision-making regarding NIPT.

The sample in this study is largely composed of white, married women with high socioeconomic status and educational attainment, and therefore does not account for how variations in race, socioeconomic status, and education may shape experiential knowledge and factor into NIPT use decision-making. Additionally, the findings of this study are specific to the U.S. context, given that the healthcare system in the U.S. is unlike that of other high-income countries in terms of the payment structure for NIPT and circumstances for use. Future studies should aim to evaluate this framework of decision-making in more diverse samples [8, 35, 36].

Finally, the frameworks outlined in this study generalize decision-making processes to an extent, and there is variation among women represented by these broad frameworks; nonetheless, they provide a useful tool for understanding how different aspects of the decision-making process tend to go together.

## Conclusion

The findings of this study highlight the iterative, dynamic nature of decision-making that is not based on static “values” and “beliefs”, but evolving embodied and empathetic experiential knowledge that shape perceptions of NIPT. Exploring the factors that contributed to significant decision-making in this sample reveals perceptions that can be used to bolster informed decision-making around NIPT use. These findings suggest that clinicians aiming to support decision-making should encourage their patients to think through how they might use the information NIPT provides and counter societal stereotypes about who should be using NIPT.

Additionally, many women in this study displayed routinized decision-making, bolstered by perceptions of common use of NIPT and technology use as progressive. Additionally, women displaying routinized uptake of NIPT expressed expectations of emotionally neutral or anxiety-relieving information and detachment between the decision to obtain information and consideration of its potential use. The observed routinization among women desiring “holistic” pregnancies suggests that routinization occurs not only in highly medicalized environments but also elsewhere due to pervasive societal perceptions. The lack of clinician influence found in this study also highlights how the factors that shape routinization can occur outside of the clinical encounter, and thus efforts to combat routinization must aim to reshape societal narratives about NIPT. This is especially important as the scope of NIPT expands to include genetic markers that are less accurately assessed with this technology.

## Supplementary information

Additional file 1: **Supplementary file 1.** Interview Guide. Questions used to guide semi-structured interviews. (TXT 3 kb)

## Data Availability

The datasets used and/or analysed during the current study are available from the corresponding author on reasonable request.

## Abbreviations

NIPT: Non-Invasive Prenatal Testing
AMA: Advanced Maternal Age
